# Standardization of rectal cancer surgery and bowel preparation in Austria: A multicenter nationwide survey by the Austrian Society of Surgical Oncology

**DOI:** 10.1007/s00508-023-02227-y

**Published:** 2023-06-26

**Authors:** Kerstin M. Widmann, Christopher Dawoud, Felix Harpain, Felix Aigner, Jaroslav Presl, Harald Rosen, Matthias Zitt, Sebastian F. Schoppmann, Klaus Emmanuel, Stefan Riss

**Affiliations:** 1 Department of General Surgery, Division of Visceral Surgery, Medical University Vienna, Waehringer Guertel 18–20, 1090 Vienna, Austria; 2 Department of Surgery, Krankenhaus der Barmherzigen Brüder Graz, Graz, Austria; 3 Department of Surgery, Paracelsus Medical University, Salzburg, Austria; 4 Department of Surgical Oncology, Sigmund Freud Private University (SFU), Vienna, Austria; 5 Department of General Surgery, Krankenhaus der Stadt Dornbirn, Dornbirn, Austria

**Keywords:** Rectal cancer, Learning curve, Transanal total mesorectal excision, Robotic surgery, Laparoscopic surgery, Bowel preparation

## Abstract

**Background:**

Standardized management of colorectal cancer is crucial for achieving an optimal clinical and oncological outcome. The present nationwide survey was designed to provide data about the surgical management of rectal cancer patients. In addition, we evaluated the standard approach for bowel preparation in all centers in Austria performing elective colorectal surgery.

**Methods:**

The Austrian Society of Surgical Oncology (ACO[“Arbeitsgemeinschaft für chirurgische Onkonlogie”]-ASSO) conducted a multicenter questionnaire-based study comprising 64 hospitals between October 2020 and March 2021.

**Results:**

The median number of low anterior resections performed annually per department was 20 (range 0–73). The highest number was found in Vienna, with a median of 27 operations, whereas Vorarlberg was the state with the lowest median number of 13 resections per year. The laparoscopic approach was the standard technique in 46 (72%) departments, followed by the open approach in 30 (47%), transanal total mesorectal excision (TaTME) in 10 (16%) and robotic surgery in 6 hospitals (9%). Out of 64 hospitals 51 (80%) named a standard for bowel preparation before colorectal resections. No preparation was commonly used for the right colon (33%).

**Conclusion:**

Considering the low number of low anterior resections performed in each hospital per year in Austria, defined centers for rectal cancer surgery are still scarce. Many hospitals did not transfer recommended bowel preparation guidelines into clinical practice.

**Supplementary Information:**

The online version of this article (10.1007/s00508-023-02227-y) contains supplementary material, which is available to authorized users.

## Introduction

Colorectal cancer (CRC) represents the third most common cancer worldwide [1]. In 2018, the incidence of CRC in Austria was 51.5 per 100,000 people, and the risk of developing CRC by the age of 75 years was 2.6%. Due to screening programs, a decrease in the incidence and mortality of CRC over the last 10 years has been observed [2].

The standard surgical treatment for rectal cancer is total mesorectal excision (TME), which results in a low incidence of local recurrence rates [3, 4]. The TME can be performed using the traditional open or minimally invasive approach, including laparoscopic, robotic and transanal techniques [5]. In a network meta-analysis, Ryan et al. found no significant differences in long-term oncological outcomes, with respect to local recurrence and survival, between all minimally invasive approaches [3].

In contrast to the traditional open surgery, the laparoscopic approach is associated with a significantly improved postoperative recovery, resulting in less pain and a shorter hospital time [6]. In comparison, the advantages of open TME were shorter operative times and more intact mesorectal specimens [6, 7]. Notably, laparoscopic TME is technically demanding, resulting in relatively high learning curves. Son et al. described a minimum of 60–80 cases to attain proficiency in conducting laparoscopic rectal resection [8]. Consequently, transanal TME (TaTME) and robotic TME have been introduced to overcome those limitations.

Patient outcome highly depends on the surgeon’s experience. Jimenez-Rodriguez et al. concluded that it was necessary to attain 21–23 cases in robotic rectal surgery to complete the learning curve [9]. Persiani et al. revealed longer learning curves for TaTME and recommended strict supervision for the first 40–50 cases [10]. In comparison, a systematic review by Burghgraef et al. showed that 32–75 cases were essential to complete the learning curve for robotic TME and 36–75 cases for TaTME [11]. Regarding Austria, it remains unknown how many rectal resections are conducted in each hospital per year and whether surgeons can achieve a sufficient number of resections to complete the learning curve and maintain a reasonable level of experience.

Bowel preparation to reduce postoperative morbidity represents another essential topic in colorectal surgery. Recent data from the American College of Surgeons National Surgical Quality Improvement Program suggested a potential role for either combined mechanical bowel preparation and preoperative oral antibiotics or oral antibiotics alone to lower the number of surgical site infections. Consequently, the American Society of Colon and Rectal Surgeons Clinical Practice Guidelines for the Use of Bowel Preparation recommended the combined approach for elective colorectal surgery. In addition, multiple studies have revealed no benefit in mechanical bowel preparation alone [12, 13]; however, it remains unclear whether surgical units in colorectal surgery follow the new recommendations.

Thus, the present nationwide survey conducted by the Austrian Society of Surgical Oncology was designed to assess the surgical management of rectal cancer patients in Austria. Additionally, we evaluated the standard approach in bowel preparation in all centers in Austria performing elective colorectal surgery.

## Methods

### Selection of hospitals

The Austrian Society of Surgical Oncology (ACO[“Arbeitsgemeinschaft für chirurgische Onkonlogie”]-ASSO) conducted this multicenter questionnaire-based study. First, we created a list of all hospitals (*n* = 101) in Austria, situated in 9 federal states, where elective colorectal surgery was performed. Private hospitals were excluded. In October 2020, the selected hospitals were contacted by email, including a link leading to a standardized questionnaire. If no response was received within 2 months, the respective surgical departments were contacted by telephone. Those who did not respond after another 2 months were called again. Finally, 64 clinics (63%) responded to the questionnaire between October 2020 and March 2021.

### Questionnaire

The survey consisted of 12 questions, 4 of which were designed to evaluate the application of bowel preparation and the remaining 8 to evaluate the performance and frequency of colorectal procedures. There were 6 single-choice questions with 2–7 options each, 1 multiple-choice question with 4 options, and 5 open-ended questions with numbers. The entire questionnaire is provided in the supplement.

### Statistical analysis

Metric variables were described using mean or median and quartiles, which were graphically represented using boxplots and histograms. Nominal variables were evaluated with frequency tables and contingency tables and specified in absolute and relative frequencies, demonstrated by bar charts. Statistical analysis was performed using the SPSS statistical software package (IBM SPSS Statistics for Mac, Version 22.0; SPSS Inc., Chicago, IL, USA).

## Results

We enrolled 64 centers, with Lower Austria having the highest number of participating departments (*n* =15, 23%, followed by Vienna, *n* = 10, 16%), and Upper Austria (*n* = 9, 14%). In Styria eight (13%) departments participated and five each (8%) in Tyrol, Salzburg and Carinthia. The lowest numbers were recorded in Vorarlberg with three (5%) and Burgenland (6%) with four centers.

### Surgeons specializing in colorectal surgery

The number of dedicated colorectal surgeons was questioned to assess the specialization grade in each hospital. In 58 out of 64 clinics (91%) surgeons specialized in the field of colorectal surgery: in 18 (31%) hospitals more than 5 surgeons performed colorectal resections, and 31 hospitals (53%) employed between 3 and 5 specialists, whereas in 8 centers (14%) precisely 2 specialists could be found. In one hospital (2%), there was only one surgeon who was authorized to perform colorectal resections. Table 1 shows the number of surgeons performing colorectal procedures in relation to the annual caseload.

**Table 1:** Median annual caseload related to the number of specialized surgeons

Number of surgeons	Number of centers	Number of rectal resections (median)
1	1	30
2	8	14
3	14	14
4	7	25
5	10	23
> 5	18	25

### Low anterior resections in Austria

The median number of low anterior resections performed annually per department was 20 (range 0–73). (Table 2) The highest number was found in Vienna, with a median of 27 operations, whereas Vorarlberg was the state with the lowest number of resections per year (*n* = 13).

**Table 2:** Median (range) number of low anterior resections (LAR) of all hospitals in every federal state. In Burgenland, no range is given because only one center answered this question

Federal state	LAR/year
Burgenland	20
Carinthia	17 (8–25)
Lower Austria	20 (0–50)
Salzburg	17 (7–50)
Styria	17 (12–55)
Tyrol	20 (15–35)
Upper Austria	23 (10–73)
Vienna	27 (15–50)
Vorarlberg	13 (10–25)
Median and range of all 9 federal states	20 (0–73)

### Approaches for TME

**Fig. 1:**
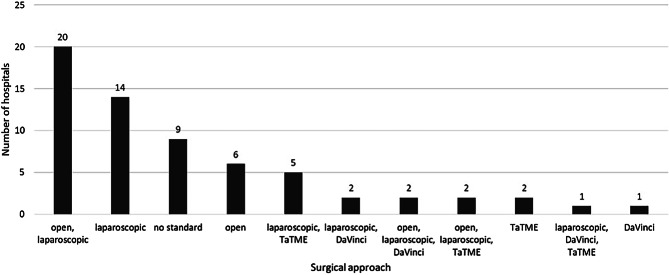
List of different options hospitals could vote for as their primary approach for rectal resection. The question allowed a multiple choice answer

The questionnaire offered multiple answers regarding the standard operating procedure for TME. Participants could select more than one procedure as their first-choice method for treating rectal cancer. The laparoscopic approach was named most frequently, with 46 (72%) of the centers naming it as their method of choice, 30 hospitals (47%) opted for a conventional open procedure as their primary approach, while TaTME was preferred in 10 (16%) departments only. Furthermore, six hospitals (9%) stated that robotic surgery was their preferred technique. The combination consisting of laparoscopic and open surgery was chosen most often by 20 out of 64 hospitals (31%). The results are visualized in Fig. 1.

### Numbers of robotic surgery and TaTME procedures in Austria

Among the hospitals that indicated DaVinci as one of their standard procedures, the highest number of operations in 1 clinic was 40 cases per year. The surgical department with the second highest number of patients treated with the robotic approach performed 20 procedures on average per year. Notably, few centers using DaVinci performed only two or three operations annually. In 6 hospitals, TaTME was performed more than 10 times per year. Interestingly, two hospitals reported having one annual operation only. (Table 3).

**Table 3:** Numbers of transanal total mesorectal excision (TaTME) and Da Vinci resections per year of all hospitals which use these procedures

TaTME	*n* /year	Da Vinci	*n* /year
Hospital I	40	Hospital I	40
Hospital II	27	Hospital II	20
Hospital III	20	Hospital III	10
Hospital IV	15	Hospital IV	10
Hospital V	12	Hospital V	8
Hospital VI	10	Hospital VI	3
Hospital VII	6	Hospital VII	2
Hospital VIII	5	–	–
Hospital IX	5	–	–
Hospital X	5	–	–
Hospital XI	4	–	–
Hospital XII	2	–	–
Hospital XIII	1	–	–
Hospital XIV	1	–	–

### Bowel preparation

Out of 64 hospitals 51 (80%) defined a standard approach for bowel preparation before colorectal resections, however, in 7 (11%) departments, there was no standard for bowel preparation, and each surgeon stated it differently. In addition, 6 (9%) hospitals did not respond to bowel preparation questions.

**Fig. 2:**
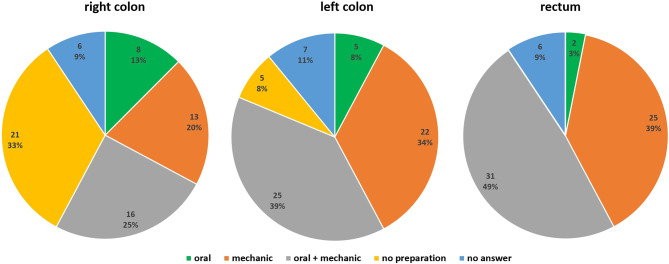
Pie charts showing the use of bowel preparation before colorectal surgery for right/left colon and rectum

For the rectum and the left colon, the combination of oral antibiotics and mechanical preparation, which refers to the administration of an oral preparation prior to surgery in order to clear the bowel of any fecal matter, was the most commonly selected option (48% and 39%), followed by using only mechanical preparation (39% and 34%). On the contrary, no preparation for the right colon and the use of a combined preparation were chosen in 33% and 25%, respectively (Fig. 2).

## Discussion

The purpose of this study was to describe the current clinical practice in Austria for standardized rectal cancer surgery. Additionally, we assessed whether bowel preparation before elective colorectal surgery reflected international recommendations for optimal patient care. We found that there are few well specialized rectal surgery centers in Austria, owing to the low number of low anterior resections performed in each hospital per year. Interestingly, a few hospitals named minimally invasive techniques such as TaTME or robotic surgery as their standard procedure for treating rectal cancer surgically. This is surprisingly taking into account that most departments perform two or three resections per year only. Given the estimated learning curves for TaTME (36–54 cases) and robotic surgery (32–75 patients), the question arises, whether the implementation in every center is reasonable and justified [11].

There has been clear evidence that the postoperative outcome after rectal resections also depends on the surgeon’s training, experience, and volume [14]. Aquina et al. indicated that the postoperative mortality and rate of permanent colostomies were significantly reduced by 57% and 35%, respectively, when rectal resections were performed in high-volume hospitals by high-volume surgeons. According to their definition, such dedicated hospitals undertook at least 25 rectal resections per year, and 1 surgeon conducted at least 10 rectal resections annually [15]. Ho et al. and Hodgson et al. revealed similar results. They demonstrated that patients who underwent rectal resection in specialized hospitals had decreased postoperative mortality and were less likely to obtain a permanent colostomy [16, 17]. In our study, 10 departments showed an annual number of 10 or fewer cases. Even surgeons with many years of experience need to achieve a certain number of procedures to maintain their surgical skills. Surgeons in training may not even complete an adequate number of operations to meet the learning curves.

Van Oostendorp et al. revealed that learning curves played a significant effect, especially for the first 10 operations, in which high rates of local recurrence and anastomotic insufficiency were noted. This was especially the case for novel techniques such as TaTME, where a substantial decrease in the local recurrence rates was observed after 40 procedures [18]. A study in Norway further questioned the safety of TaTME as they found unfavorable rates of local recurrence and anastomotic leakage compared to the Norwegian Colorectal Cancer Registry [19]. In line with other countries, Switzerland established centers for highly specialized medicine, where rectal resections are performed routinely. A caseload of at least 25 resections per year was defined as a cut-off number [20]. A study by Kaech et al. showed that this process of centralization had a positive effect on postoperative mortality [20].

The other main topic of our questionnaire addressed the standardization of bowel preparation regimens among Austrian hospitals. Notably, the majority of participating facilities (84%) reported using a standard preparation technique prior to elective colorectal resections; however, the protocols differed significantly between hospitals and were not always in line with current guideline recommendations. For example, mechanical bowel preparation alone for the right colon was conducted in 20% and for the left colon in 34%. Taking into account the current evidence, mechanical preparation alone is not suggested in colonic surgery, as a reduction in anastomotic leakage rates and surgical site infections was not observed in contrast to no preparation [13]. Interestingly, a European survey by Devane et al. revealed that mechanical bowel preparation alone was routinely prescribed by about 30% of the responders before colon surgery, which was similar to our results [21].

Several surveys [22–24] showed similar results with infrequent use of oral antibiotics and regular use of mechanical bowel preparation, despite a significantly lower incidence of surgical site infections when using a combination of both [25]. On the contrary, data from the USA revealed a more common use of a combined preparation [26]. This was also in the line with the present results, where a combined preparation before rectal resection was observed in almost 50%.

There were a few limitations of the study that need to be addressed. First, although we provided a large number of participating departments, it does not include all hospitals in Austria. It is also worth mentioning that all numbers were individually entered and calculated by each participating hospital but were not confirmed by national statistics.

## Conclusion

Considering the low number of anterior resections conducted in each hospital annually, specialized departments for rectal cancer surgery are still lacking in Austria. Notably, many hospitals did not follow guideline recommendations for bowel preparation routinely.

## Supplementary Information

The entire questionnaire about rectal cancer surgery and bowel preparation is provided in the supplements.
