# Supplementary material for: Standardization of rectal cancer surgery and bowel preparation in Austria: A multicenter nationwide survey by the Austrian Society of Surgical Oncology
Source: Wien Klin Wochenschr. 2023 Jun 26;135(17-18):457–62. doi: 10.1007/s00508-023-02227-y (PMC10497700; doi:10.1007/s00508-023-02227-y)
Supplement: Supplementary file 1 — The entire questionnaire about rectal cancer surgery and bowel preparation is provided in the supplements. [file 508_2023_2227_MOESM1_ESM.docx]

**Supplement**

| Are there dedicated surgeons for colorectal surgery at your hospital? | - Yes - No | Single choice answer |
| --- | --- | --- |
| How many surgeons perform colorectal resections? | - 0 - 1 - 2 - 3 - 4 - 5 - >5 | Single choice answer |
| How many low anterior resections are performed annually at your department? |  | Open answer |
| What is your standard operating procedure for TME? | - Open - Laparoscopic - Robotic - TaTME | Multiple choice answer |
| How many open rectal resections are performed annually at your department? |  | Open answer |
| How many laparoscopic rectal resections are performed annually at your department? |  | Open answer |
| How many Da Vinci rectal resections are performed annually at your department? |  | Open answer |
| How many TaTME rectal resections are performed annually at your department? |  | Open answer |
| Does your department have a standard bowel preparation prior to colorectal surgery? | - Yes - No | Single choice answer |
| Type of preferred bowel preparation for the right colon? | - None - Mechanical preparation - Mechanical + oral preparation - Oral preparation | Single choice answer |
| Type of preferred bowel preparation for the left colon? | - None - Mechanical preparation - Mechanical + oral preparation - Oral preparation | Single choice answer |
| Type of preferred bowel preparation for the rectum? | - None - Mechanical preparation - Mechanical + oral preparation - Oral preparation | Single choice answer |
